# Factors Associated with Maternal Engagement in Infant Care When Mothers Use Substances

**DOI:** 10.1089/whr.2022.0082

**Published:** 2023-02-06

**Authors:** Kalyn M. Renbarger, Barbara Phelps, Allyson Broadstreet, Sheila Abebe

**Affiliations:** School of Nursing, Ball State University, Muncie, Indiana, USA.

**Keywords:** maternal, engagement, NAS, substance use

## Abstract

**Introduction:**

Mothers who use substances can play a key role in the treatment and care of their infants. However, challenges exist to engaging these mothers in the care of their infant. The purpose of this study was to identify factors associated with maternal engagement in infant care when mothers are experiencing substance use disorders.

**Materials and Methods:**

A systematic search was conducted using the databases of CINAHL, APA PsycINFO, and PubMed along with a manual search of Google Scholar between the years of 2012 and 2022. Studies were included if they were (1) original qualitative research; (2) published in English; (3) peer reviewed; (4) from the perspective of mothers who use substances or nurses; (5) included descriptions of interactions between mothers who use substances and their infants during postpartum care, and/or in the nursery or neonatal intensive care unit; and (6) conducted in the United States. The studies were assessed for quality and validity using 10 criteria from the Joanne Briggs Institute critical appraisal checklist for qualitative research.

**Results:**

Findings from 22 qualitative studies were synthesized using a thematic synthesis approach and revealed 3 overarching themes that included 7 descriptive subthemes that identified factors to maternal engagement. The seven descriptive subthemes included: (1) Attitudes Toward Mothers Who Use Substances; (2) Knowledge on Addiction; (3) Complicated Backgrounds; (4) Emotional Experiences; (5) Managing Infant Symptoms; (6) Model of Postpartum Care; and (7) Hospital Routines.

**Discussion:**

Participants described stigma from nurses, complex backgrounds of mothers who use substances, and postpartum models that influenced mothers' engagement in infants' care. The findings suggest several clinical implications for nurses. Nurses should manage their biases and approach mothers who use substances in a respectful manner, increase their knowledge of issues and care related to addiction in the perinatal period, and promote family-centered approaches to care.

**Conclusion:**

The findings of 22 qualitative studies described factors associated with maternal engagement in mothers who use substances that were integrated using a thematic synthesis method. Mothers who use substances have complex backgrounds and experience stigma which can negatively impact their engagement with their infants.

## Background

### Maternal substance use

Maternal substance use is a growing concern in the United States and has been linked to poor maternal–infant health outcomes. Women living in the United States have reported tobacco use (11.6%), alcohol use (9.9%), and illicit drug use (5.4%) during pregnancy.^1^ The opioid epidemic has resulted in rising rates of maternal use during pregnancy. Between 2010 and 2017, the rate of maternal opioid-related diagnoses at the time of delivery had increased 131% from 3.5 to 8.2 per 1000 delivery hospitalizations.^2^ In addition, 6.6% of pregnant women in the United States used prescription opioids in 2019.^3^

Substance use has been identified as a leading contributor to maternal deaths.^4–6^ Other complications include prematurity, placental abruption, stillbirth, increased infant mortality, low birth weight, oral clefts, clubfoot, neonatal opioid withdrawal syndrome (NOWS), and neonatal abstinence syndrome (NAS).^7,8^ NAS is a collection of withdrawal symptoms in newborns who have been exposed to substances. Rates of NAS have increased rapidly in the United States in recent years. From 2010 to 2017, rates of NAS in 47 states and District of Columbia increased 82% from 4.0 to 7.3 birth hospitalizations.^2^ Symptoms include irritability, tremors, poor feeding, respiratory issues, gastrointestinal difficulties, and a variety of other symptoms.^9^ A subset of NAS is neonatal opioid withdrawal syndrome (NOWS) and comprises withdrawal symptoms specifically from prenatal opioid use.

The most frequently used tool to assess the severity of NAS symptoms in the United States is the Modified Finnegan Neonatal Abstinence Scoring System.^10^ This tool was developed to assess withdrawal symptoms in infants exposed to substances prenatally and is composed of 21 different criteria regarding central nervous system, metabolic, vasomotor, respiratory, and gastrointestinal disturbances. Infants with a score of 8 or higher are considered to have more severe withdrawal symptoms and may require pharmacologic intervention.^10^ Mild cases of NAS may be managed solely by nonpharmacological interventions, while more severe symptoms of NAS may require medication and result in increased length of hospital stays and higher acuity of care. Methods of nonpharmacological inventions include swaddling, positioning, quiet and dimly lit rooms, minimal stimulation, rooming-in, skin-to-skin contact, breastfeeding, and infant positioning.^11^

Mothers who use substances have played a significant role in managing symptoms of NAS in their infants. Prior literature has demonstrated that rooming-in (a postpartum care model where the infant remains in the same hospital room as the mother), breastfeeding, and having a constant caregiver decreases the severity of symptoms of NAS, length of hospitalization, and need for pharmacological treatment in infants with NAS.^12^ Nurses working in perinatal settings are responsible for assisting mothers who use substances to engage in nonpharmacological interventions and care of their infants. However, both nurses and mothers who use substances have reported challenges to maternal engagement in infant's care. Stigma may be a contributing factor to maternal engagement in infant care.

It has been cited that nurses and other health care providers view substance use as a moral failing rather than a medical condition and their judgments can interfere with appropriate infant care.^13^ Mothers who use substances have also reported difficulty engaging with their infants due to the severity of NAS symptoms (*i.e.*, inconsolable crying, stiffness) and the disruptive assessments related to the scoring of NAS symptoms.^14^

While several qualitative studies exist to describe factors associated with maternal engagement in mothers who use substances, to our knowledge, these studies have not been well-synthesized. Nuanced descriptions of factors associated with maternal engagement in infant's care are needed to develop strategies to be used by nurses to enhance the care of mothers who use substances and their infants.

## Materials and Methods

Research studies were eligible for inclusion if they were (1) original qualitative research; (2) published in English; (3) peer reviewed; (4) from the perspective of mothers who use substances in the perinatal period or from the nursing perspective; (5) included descriptions of engagement between mothers who use substances and their infants during postpartum care, and/or in the nursery/neonatal intensive care unit (NICU); (6) conducted in the United States; and (7) published no earlier than January 1, 2012. The term nurse was used broadly and included the following terms to capture the nursing perspective: nurse, nurse practitioner, nursing assistant, and midwives. Studies were limited to qualitative studies conducted in the United States because of immense differences in culture and norms in health care settings that exist worldwide.

The research studies were retrieved through the databases of APA PsycINFO, CINAHL, and PubMed. A search term for mother in the perinatal period (*e.g.*, maternal, perinatal) or nurse (*e.g.*, nurse, midwives) was combined with a term to capture substance use (*e.g.*, substance use, NAS), a term to capture qualitative research (*e.g.*, qualitative, narrative), and a term to capture infant care experiences (*e.g.*, experience, engage). Using this method, the following search terms were exhausted: maternal, perinatal, women, perinatal, postpartum, NICU, mother, substance use, NAS, opioid, illicit, methadone, buprenorphine, smoking, nurse, midwives, nurse practitioner, qualitative, narrative, phenomenology, grounded theory, ethnography, experience, interaction, engagement, involve, participation, breastfeeding, kangaroo care, infant feeding, and rooming-in. A manual search of Google Scholar using various combinations of the above search terms was also conducted to identify inclusion studies that may have been missed in the systematic search.

Members of the research team reviewed the studies by their titles.

Studies were eliminated if the title did not contain a search term for women in the perinatal period and a term for substance use. Next, the remaining studies were reviewed by their abstract and eliminated if they did not meet the inclusion criteria.

### Quality assessment and data extraction

Ten criteria from the Joanne Briggs Institute (JBI) critical appraisal checklist for qualitative research were used to assess each of the studies for credibility and quality.^15^ Consensus among the authors was reached through discussion. The third author then created a cross-study display table that provided characteristics of the inclusion studies (Appendix Table A1). Data describing factors associated with mothers who use substances participating in their infant's care were extracted and reviewed. A thematic synthesis described by Thomas and Harden.^16^ was used to integrate the findings of the included studies. The three stages of thematic synthesis highlighted by Thomas and Harden^16^ include the following: (1) free line-by-line coding of the findings of the original studies, (2) the development of the free codes into associated areas to construct descriptive subthemes, and (3) the development of overarching analytical themes.

Descriptive subthemes stay close to the data in the original studies, whereas analytical themes build higher levels of abstraction to produce new interpretive concepts.^16^ The research team for this study consisted of two doctorally prepared nurse researchers with qualitative and maternal–child health expertise, a master's prepared nurse certified in maternal–child nursing, and a graduate student in a psychology program. First, the researchers independently reviewed each included study. Second, the first author individually used line-by-line coding of the extracted data and organized key components that described factors related to mothers who use substances participating in their infant's care. Third, the first and second authors viewed together for similarities and differences between the codes.

There were a few differences of opinion that were resolved through discussion and returning to the original study. Fourth, the first and second authors organized the identified key components into descriptive subthemes. Finally, the first and second authors analyzed the descriptive subthemes to move beyond the findings of the original studies as outlined by Thomas and Harden.^16^ During this process, new overarching synthesis themes were discovered.

## Results

**FIG. 1.**
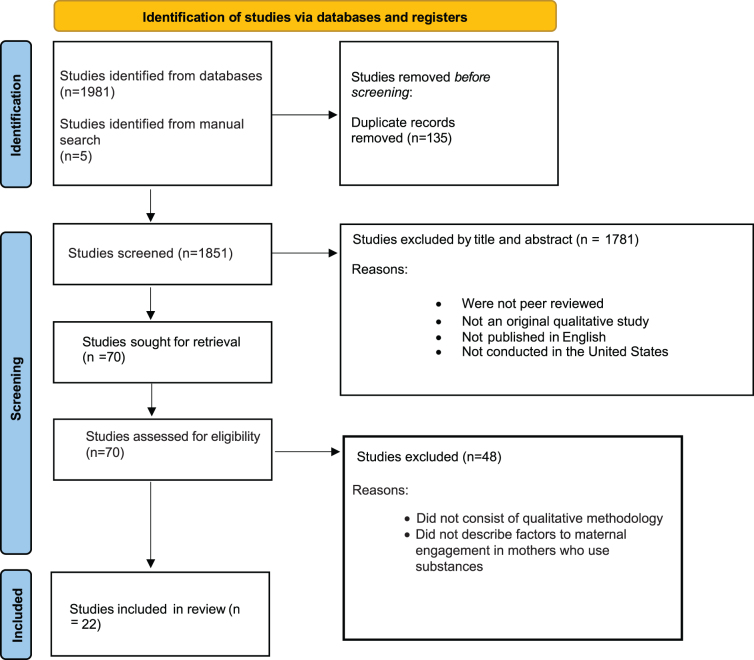
Preferred Reporting Items for Systematic Reviews and Meta-Analyses diagram of systematic search results.

The databases of APA PsycINFO, CINAHL, and PubMed were searched between January 2022 and March 2022 and resulted in 1981 studies for review. A manual search of Google Scholar resulted in an additional five studies. After removing duplications, 1851 studies remained. Of the remaining 1851 studies, 1683 were removed by title and 98 were removed by abstract for not meeting the inclusion criteria, thus leaving 70 for full review. Of the 70 studies, 48 were removed for not using qualitative methodology and did not describe factors associated with maternal engagement. Thus, 22 studies were included in this review. Results of the systematic search are displayed using the Preferred Reporting Items for Systematic Reviews and Meta-Analyses (PRISMA) Diagram of Systematic Search Results (Fig. 1).

The sample sizes of each study ranged from 5 to 67 participants. Each of the studies used a qualitative method, which included one study that used a mixed-method approach. Although most of the studies were descriptive in nature, two studies used a focused ethnography, and one used a grounded theory approach.

Seven descriptive subthemes were identified in the analysis and include the following: (1) *Attitudes Toward Mothers Who Use Substances*, (2) *Knowledge on Addiction*, (3) *Complicated Backgrounds*, (4) *Emotional Experiences*, (5) *Managing Infant Symptoms*, (6) *Model of Postpartum Care*, and (7) *Hospital Routines*. Three overarching synthesis themes emerged to describe factors associated with maternal engagement and included the following: (1) *Nursing Characteristics Influencing Engagement;* (2) *Maternal Characteristics Influencing Engagement*; and (3) *Hospital Characteristics Influencing Engagement*. The descriptive subthemes and overarching synthesis themes and their contributing studies are displayed in Table 1.

**Table 1.** Overarching and Descriptive Themes: Factors to Maternal Engagement in Infant Care

Descriptive subthemes	Contributing studies
Overarching synthesis theme 1: *Nursing Characteristics Influencing Engagement*	
1. *Attitudes Toward Mothers Who Use Substances*	Adrian et al,^35^ Atwood et al,^33^ Busse et al,^36^ Cleveland and Bonugli,^37^ Cleveland et al,^38^ Cleveland and Gill,^39^ Demirci et al,^40^ Fallin-Bennett and Ashford,^41^ Howard et al,^42^ Loyal et al,^43^ McGlothen-Bell et al,^44^ Nelson,^45^ Reese et al,^46^ Rockefeller et al,^47^ Shuman et al,^48^ Shuman et al^49^
2. *Knowledge on Addiction*	Adrian et al,^35^ Busse et al,^36^ Carlson and Kieran,^50^ Cleveland and Bonugli,^37^ Leiner et al,^51^ McGlothen et al,^52^ Reese et al,^46^ Shuman et al,^48^ Shuman et al^49^
Overarching synthesis theme 2: *Maternal Characteristics Influencing Engagement*	
3. *Complicated Backgrounds*	Adrian et al,^35^ Busse et al,^36^ Carlson and Kieran,^50^ Cleveland and Bonugli,^37^ Cleveland et al,^38^ Fallin-Bennett and Ashford,^41^ Howard et al,^42^ Kramlich et al,^53^ Leiner et al,^51^ Shuman et al^48^
4. *Emotional Experiences*	Carlson and Kieran,^50^ Cleveland and Bonugli,^37^ Cleveland et al,^38^ Demirci et al,^40^ Howard et al,^42^ Kramlich et al,^53^ Leiner et al,^51^ Maguire et al,^54^ McRae et al,^34^ Rockefeller et al^47^
5. *Managing Infant Symptoms*	Cleveland et al,^38^ Howard et al,^42^ Loyal et al,^43^ Maguire et al,^54^ Rockefeller et al^47^
Overarching synthesis theme 3: *Hospital Characteristics Influencing Engagement*	
6. *Model of Postpartum Care*	Atwood et al,^33^ Cleveland et al,^38^ McGlothen-Bell et al,^44^ Howard et al,^42^ Kramlich et al,^53^ Reese et al,^46^ Shuman et al^49^
7. *Hospital Routines*	Atwood et al,^33^ Howard et al,^42^ Loyal et al,^43^ McGlothen-Bell et al,^44^ McRae et al,^34^ Rockefeller et al,^47^ Shuman et al^49^

### Nursing characteristics influencing engagement

These studies described characteristics of nurses that influenced maternal engagement in infant's care. *Nursing Characteristics Influencing Engagement* included two descriptive subthemes that informed the overarching synthesis theme.

### Attitudes toward mothers who use substances

Sixteen studies contributed to the descriptive subtheme of *Attitudes Toward Mothers Who Use Substances*. In these studies, nurses' judgments and biases toward the mothers who use substances served as barriers to maternal engagement in infant care. Mothers who use substances in these studies often perceived judgment from their nurses and thus avoided visiting their infants, breastfeeding, and participating in skin-to-skin care as the result of their interactions with their nurses. Some nurses in these studies viewed mothers who use substances as barriers to their infants' care and viewed working with the mothers as a futile effort. As a result, mothers who use substances felt left out or avoided caring of their infants.

Conversely, facilitators to maternal engagement included nurses who were able establish trust such as through validating emotions, communicating objectively and honestly, and empowering mothers who use substances to engage in their infants' care. Some nurses stressed the importance of being nonjudgmental to prevent the mothers from having “a wall” go up that makes communication and promoting engagement difficult.

### Knowledge on addiction

Ten studies contributed to the descriptive subtheme of *Knowledge on Addiction*. In these studies, nurses often lacked information they needed to effectively engage mothers who use substances in the care of their infants. Nurses acknowledged not having enough knowledge and suggested that on-the-job training and education on addiction and recovery would increase their confidence in engaging mothers who use substances in the care of their infants. Similarly, mothers who use substances described not receiving enough health education on topics including skin-to-skin care, hepatitis C, and breastfeeding. As a result of receiving insufficient education, mothers who use substances sometimes felt defeated and discouraged to participate in the care of their infants.

On the contrary, mothers who use substances appreciated the helpful information provided by nurses about the care of their infant and as a result felt more prepared. The health information received by nurses and other health care providers empowered mothers who use substances to engage in the care of their infants.

### Maternal characteristics influencing engagement

Participants described characteristics of mothers who use substances that influenced their engagement with their infants. *Maternal Characteristics Influencing Engagement* included three descriptive subthemes that informed the overarching synthesis theme.

### Complicated backgrounds

Ten studies contributed to the descriptive subtheme of *Complicated Backgrounds*. In these studies, challenges in the daily lives of mothers who use substances were recognized by mothers who use substances and nurses. These studies highlighted that mothers who use substances often have histories of trauma, difficulties in relationships, and other children at home, which served as barriers to engagement with their infants. Mothers who use substances believed their providers and nurses did not always understand their situations such as not being able to be present due to caring for other children. Mothers who use substances expressed a desire for nurses to display more understanding about their complex situations. Nurses also expressed concerns over the complexity of the mothers' lives and their ability to care for their infant after hospital discharge.

### Emotional experiences

Ten studies contributed to the descriptive subtheme of *Emotional Experiences*. In these studies, mothers who use substances often experienced emotional distress watching their infants withdrawing from prenatal substance use and sometimes avoid engaging with their infants. For example, some mothers expressed not wanting to visit the NICU because of the guilt and pain experienced when seeing their infant's symptoms of withdrawal. Other mothers who use substances were fearful of harming their infants through breastfeeding and as a result decided not to breastfeed. Nurses in these studies also detected emotional distress from mothers who use substances and often felt the necessity to help and teach them how to relax.

Contrarily, mothers who use substances sometimes described bonding and joyful emotions that served as motivation to engage with their infants. Mothers who use substances believed that engaging with their infants through skin-to-skin contact and feeding brought on a strong emotional and physical connection to their infant. As a result, being engaged in their infants' care helped mothers who use substances atone for the consequences of their substance use.

### Managing infant symptoms

Five studies contributed to the descriptive subtheme of *Managing Infant Symptoms*. In these studies, mothers who use substances described challenges they had when their infants experienced symptoms of NAS including feeding difficulties and inconsolable crying. Mothers who use substances discussed the stress and feelings of inadequacy when they were unable to console their infant. Mothers who use substances reported crying when observing their infants having tremors and irritability. Nurses in these studies confirmed that consoling and feeding infants with NAS were challenging for mothers who use substances. Nurses described times when mothers who use substances preferred infants to be medicated when seeing withdrawal rather than trying other nonpharmacological methods first, which would involve their engagement with their infants.

### Hospital characteristics influencing engagement

Participants described hospital characteristics that influenced maternal engagement. *Hospital Characteristics Influencing Maternal Engagement* included two descriptive subthemes that informed the overarching synthesis theme.

### Model of postpartum care

Seven studies contributed to the descriptive subtheme of *Model of Postpartum Care*. In these studies, participants described challenges to maternal engagement when the mothers were unable to room-in with their infants at the hospital. Mothers who use substances often described logistical barriers to visiting their infants such as having transportation and food. Mothers who use substances also described the physical exhaustion from traveling back and forth to visit their infant at the hospital. The lack of privacy in the nursery sometimes prevented mothers who use substances in engaging with their infants such as through skin-to-skin care and breastfeeding.

Nurses also felt restricted due to privacy issues on what information they could give mothers who use substances in the nursery. Conversely, nurses believed that mothers who use substances who were able to room-in with their infant promoted maternal engagement. Nurses described mothers who use substances as assuming full care of their infant when they were able to room-in with their infant.

### Hospital routines

Seven studies contributed to the descriptive subtheme of *Hospital Routines*. In these studies, participants described hospital routines that were often inconsistent and unpredictable, which hindered their ability to engage with their infants. Some of these routines included nursing assessments, specimen collections, and medication administration. Mothers who use substances also explained how nurses entering and exiting the room interfered with their ability to relax when caring for their infant. Mothers who use substances also reported that the frequent and strict NAS scoring schedule would interrupt their abilities to feed, comfort, and nurture their infants. The mothers who use substances believed that the NAS scoring of their infants was disruptive and made their infants irritable, which increased the appearance that the infants were experiencing more severe NAS symptoms. Nurses in these studies reiterated about the disruptive nature of having to disturb the infants to perform NAS scoring.

Conversely, hospital routines with fewer interventions and that normalized newborn care were supported by mothers who use substances. Mothers who use substances enjoyed flexible hospital routines where they were encouraged to engage through holding, comforting, and feeding their infant.

## Discussion

The thematic synthesis resulted in 22 studies that described factors associated with maternal engagement in mothers who use substances. The overarching synthesis themes and descriptive subthemes identified in this study suggest that stigma from nurses served as a barrier to maternal engagement with their infant. This concept echoes other studies where nurses have expressed their frustrations and biases toward mothers who use substances.^14,17^ Mothers who use substances have also reported feeling judged by nurses and other health care providers.^18,19^ Stigma from nurses contributed to mothers who use substances emotional distress. In another study, nurses reported mothers “stop trying” when they feel judgment from nurses.^14^ Conversely, nurses and other health care providers who were caring, understanding, and affirming toward mothers who use substances were better poised to facilitate trusting relationships and improve health outcomes.^20^

In a review of literature, Kramlich et al^21^ linked positive maternal and neonatal health outcomes to engagement in prenatal care and caring relationships with health care providers. In prior literature, mothers who use substances have attributed a supportive health care provider as key in making the decision to initiate and continue breastfeeding their infants.^18,22^

Despite the benefits of breastfeeding that include the reduction of NAS symptoms and decreased length of hospital stay,^23^ mothers who use substances described many challenges to breastfeeding their infants. For example, mothers who use substances often have complex histories of trauma resulting from childhood maltreatment and sexual abuse, which participants in this study identified as a barrier to breastfeeding. This finding supports a review of literature where childhood maltreatment was associated with decreased and shorter duration of breastfeeding.^24^ In addition, mothers who use substances in the inclusion studies believed nurses to be uninformed of addiction-related issues particularly as it relates to breastfeeding and discouraged them from breastfeeding their infants. This finding resonates with previous literature on breastfeeding as it relates to mothers who use substances.

In a review of literature, Holmes et al^25^ explained that a significant barrier to breastfeeding exists as a result of inaccurate and inconsistent information received by mothers who use substances from health care providers.

Hospital environments where mothers who use substances could not room-in with their infants and experienced disruptive hospital routines served as a barrier to maternal engagement. This resounds with other studies supporting the use of rooming-in of mothers who use substances with their infants. In a review of literature, MacVicar and Kelly^26^ discovered that rooming-in was rated favorably by mothers who use substances and increased breastfeeding initiation versus other models where mothers were separated from their infants. Rooming-in was also found to increase the likelihood that mothers who use substances would be discharged from the hospital with custody of their infants. Rooming-in also been shown to increase maternal sense of competence in care of the infant.^27^

### Clinical implications

The findings of this study suggest several clinical implications for nurses, health care organizations, and researchers. First, nurses should consider that many mothers who use substances have complex histories and trauma and care for them in an empathetic, compassionate, and respectful manner.

The Association of Women's Health, Obstetric and Neonatal Nursing (AWHONN) has addressed unjust treatment in maternal–child health by establishing guidelines to support respectful maternity care for all women using the *Respectful Maternity Care Framework* (RMC).^28^ Respectful maternity care consists of basic rights to ensure that women receive maternity care that is free of abuse and disrespect.^28^ The RMC guides the provision of institutional policies. Overall goals of the guidelines are to promote actions to be implemented to promote a culture of respect for all women at all times and in all settings.^28^ Using the RMC, nurses can include mothers who use substances in health decisions and provide them with adequate health information to properly care for their infants.

Next, nurses need to acknowledge and confront personal biases they may have toward mothers who use substances and approach these mothers in a nonjudgmental and respectful manner. Nurses need to understand how judgments against mothers who use substances negatively impact the emotional response of these mothers who use substances and contribute to their challenges with maternal engagement. Health care organizations that mandate bias training for nurses and other health care providers endorse the message that equity, respect, and accountability of health care organizations are necessary to advance equity for all individuals.^29^

These findings also suggest that health care organizations should provide training to nurses on evidenced-based practices for care of mothers who use substances and their infants. Efforts should be made to avoid the separation of mothers who use substances and their infants and to offer rooming-in, when possible, to promote maternal engagement and breastfeeding. Nurses should also be trained to identify which mothers who use substances are candidates for breastfeeding and which are not. Health care organizations should consider a trauma-informed care model to fully support mothers who use substances who are eligible to breastfeed their infants.^30^ A trauma-informed approach to breastfeeding support is necessary to promote the mental health of mothers who use substances and optimal breastfeeding.

Health care organizations can adopt the Substance Abuse and Mental Health Services Administration's (SAMHSA)^30^ six key principles to a trauma-informed approach, which include the following: (1) safety, (2) trustworthiness and transparency, (3) peer support, (4) collaboration and mutuality (5) empowerment, voice, and choice; and be sensitive to (6) cultural, historical, and gender issues.

Lastly, health care organization can implement the use of family-centered approaches to overcome barriers to maternal engagement in their infant's care. One family-centered approach referred to as Eat, Sleep, Console (ESC) focuses on the comfort and care of infants with NAS by using nonpharmacologic methods. The ESC encourages the involvement of mothers who use substances and other family members in the treatment of infants.^31^ The ESC promotes improved quality of caregiving. Assessments are typically done after the infant has eaten and preferably while the infant is being held, skin-to-skin, or swaddled by their mother, resulting in a less invasive approach.^31^ This less invasive approach may assist mothers who use substances in consoling their infants and promote maternal–infant bonding by decreasing the emotional distress of the mothers who use substances and providing a less disruptive environment.

The ESC approach has been shown to decrease the need for pharmacological treatment in infants with NAS and decrease their length of hospital stay.^32^ Family-centered approaches may be significant in improving long-term health outcomes for infants with NAS and their families.

### Limitations

The findings of this study should be considered in the context of limitations. In two inclusion studies,^33,34^ other family members were interviewed along with the mothers who use substances and some data may not have been solely from the perspective of the mothers. However, the amount of data is small and mothers who use substances in other studies expressed similar thoughts, resulting in the decision to include the studies. It could not be determined if differences exist in mothers who use substances abilities to engage with their infant depending on what type of substances they used during their pregnancy (*e.g.*, licit and illicit). Future research is needed with larger and more diverse samples.

## Conclusion

The findings of 22 qualitative studies described factors associated with maternal engagement in mothers who use substances that were integrated using a thematic synthesis method. We identified three overarching synthesis themes and seven descriptive subthemes. Study participants described the characteristics of nurses, mothers who use substances, and the hospital environment that influenced maternal engagement. Stigma from nurses, the complex backgrounds of mothers who use substances, and models of postpartum care influenced maternal engagement. Nurses who work with mothers who use substances should confront their biases and approach these mothers in a respectful manner, increase their knowledge on evidenced-based practices on caring for mothers who use substances and their infants, and promote family-centered approaches to care.

## Abbreviations Used

ESC: Eat, Sleep, Console
JBI: Joanne Briggs Institute
NAS: neonatal abstinence syndrome
NICU: neonatal intensive care unit
NOWS: neonatal opioid withdrawal syndrome
OUD: opioid use disorder
RMC: *Respectful Maternity Care Framework*
SUD: substance use disorders

## Appendix

**Appendix Table A1.** Characteristics of Inclusion Studies

Author(s)	Purpose statement	Sample/setting	Method	Procedures	Findings
Adrian et al^A1^	“To investigate barriers and enablers to nurses' implementation of nonpharmacological interventions for infants with neonatal abstinence syndrome through the lens of the Theoretical Domains Framework”	Sample: 56 nurses (15 for qualitative portion of study) Setting: United States	Mixed methods	Qualitative data were collected through semistructured individual interviews. These interviews were recorded and transcribed, and transcriptions were analyzed for content.	Enablers and barriers to nurses' implementation of nonpharmacological interventions included: 1. Education 2. Experience 3. Ability to implement nonpharmacological interventions 4. Parental participation 5. Stigmatization 6. Lack of managerial/organizational support 7. Staffing ratios 8. Internal and external resources 9. Stress
Atwood et al^A2^	“We interviewed families to understand their experiences during the newborn hospitalization for NAS to improve family-centered care.”	Sample: 20 families affected by NAS Setting: United States	Qualitative	Family members were interviewed *via* phone or in-person. Interviews were recorded, transcribed, and analyzed for content.	Five domains of family experience were identified: 1. Parents' desire for education about the course and treatment of NAS 2. Parents valuing their role in the care team 3. Quality of interactions with staff (supportive versus judgmental) and communication regarding clinical course 4. Transfers between units and inconsistencies among providers 5. External factors such as addiction recovery and economic limitations
Busse et al^A3^	“To engage nurses, with experience caring for pregnant and postpartum individuals with OUD, in priority setting as a way to identify areas of need in the current health care system”	Sample: 67 nurses at an Association for Women's Health Obstetric and Neonatal Nursing Setting: United States	Qualitative	In phase 1, participants were encouraged to submit questions for phase 2 focus group. Phase 2: Focus group selected priority questions. Focus group was conducted, recorded, and transcribed. Transcriptions were analyzed for content.	Participants prioritized the following: 1. Funding to support improvements in OUD care in the perinatal period 2. Increased access to services 3. Supportive housing for individuals in recovery 4. Standardization of care 5. Efforts to destigmatize care
Carlson and Kieran^A4^	“To describe the way the mothers with opioid use disorder cana be supported to care for their newborns.”	Sample: 26 nurses and parents Setting: United States	Qualitative	Semistructured interviews were conducted and recorded. Recordings were transcribed and analyzed for content.	Nine themes were identified: 1. Developing OUD 2. Trauma/disrupted relationships 3. Violence 4. Incoherent birth stories 5. Incarceration 7. Homelessness 8. Relationships with nurses 9. Attachment and bonding
Cleveland and Bonguli^A5^	“To describe the experiences of mothers of infants with neonatal abstinence syndrome (NAS) in the neonatal intensive care unit (NICU)”	Sample: 15 mothers of infants with NAS outpatient addiction treatment facilities Setting: United States	Qualitative	Semistructured interviews were conducted with the subject, interviews were then transcribed and analyzed for content and themes.	Four themes were identified: 1. Understanding addiction 2. Watching the infant withdraw 3. Judging 4. Trusting the nurses
Cleveland et al^A6^	“To describe the mothering experiences of women with substance use disorders”	Sample: 15 mothers who had experienced an SUD during pregnancy in two community-based treatment facilities Setting: United States	Qualitative	Semistructured interviews were conducted and audio-recorded. Recordings were transcribed and analyzed for content.	Five themes that described the mothering experiences of women with SUD were identified: 1. Facing the reality of a pregnancy complicated by substance use, trauma, and loss 2. Finding a higher meaning 3. Dealing with the consequences 4. Managing the details of daily life 5. Looking toward a future with my children
Cleveland and Gill^A7^	“To describe the hospital experiences of mothers who give birth to substance-exposed infants”	Sample: 5 women who were recovering from SUD who had given birth to an infant who was admitted to the NICU Setting: United States	Qualitative	Semistructured interviews were conducted and recorded. Recordings were transcribed and analyzed for content.	Four themes were identified: 1. “Try not to judge” 2. “Scoring the baby” 3. “Share with me” 4. “I'm the mother here”
Demirci et al^A8^	“Describe[s] the perceptions surrounding breastfeeding decisions and management among pregnant and postpartum women taking methadone”	Sample: 7 pregnant women and 4 postpartum women enrolled in methadone maintenance programs Setting: United States	Qualitative	Semistructured interviews and focus groups were conducted and audio-recorded. Recordings were then transcribed, analyzed for content, and coded based on content analysis.	Three major content categories were identified: 1. Fears, barriers, and misconceptions about breastfeeding while taking methadone 2. Motivation and perceived benefits of breastfeeding 3. Sources of information, support, and anxiety about general breastfeeding management and breastfeeding while taking methadone
Fallin-Bennett and Ashford^A9^	“To collect formative information to design a tailored tobacco treatment intervention for women with newborns treated or evaluated for neonatal abstinence syndrome and to explore current tobacco use behaviors and facilitators and barriers to smoking cessation”	Sample: 11 mothers whose infants who had or were suspected to have NAS at birth within the last three months at a large academic hospital Setting: United States	Qualitative	Individual interviews were conducted and transcribed, and transcriptions were analyzed for content and themes.	Five themes were identified: 1. Strategizing to reduce risk 2. Desire to quit smoking in the future 3. Holding on to smoking while working through recovery 4. Feeling judged by nurses 5. Feeling supported and empowered by nurses
Howard et al^A10^	“To investigate perspectives of mothers with opioid use disorder regarding breastfeeding and rooming-in during the birth hospitalization and identify facilitators and barriers”	Sample: 25 mothers with opioid use disorder Setting: United States	Qualitative, grounded theory	In-depth qualitative interviews were conducted. Grounded theory analysis was used until thematic saturation was reached. Findings were triangulated, with experts in the field and a subset of informants themselves, to ensure data reliability.	Seven themes were identified: 1. Information drives maternal feeding choice 2. The hospital environment is both a source of support and tension for mothers exerting autonomy in the care of their infants 3. Opioid withdrawal symptoms negatively impact breastfeeding 4. Internal and external stigma negatively impact mothers' self-efficacy 5. Mothers' histories of abuse and trauma affect their feeding choice and bonding 6. Mothers' recovery makes caring for their infants emotionally and logistically challenging 7. Having an infant is a source of resilience and provides a sense of purpose for mothers on their path of recovery
Kramlich et al^A11^	“To understand the experience of accessing care necessary for substance use disorder recovery, pregnancy, and parenting”	Sample: 13 mothers with histories of substance use Setting: United States	Qualitative, focused ethnography	Semistructured interviews were conducted and recorded. Recordings were transcribed and analyzed for content. Observations were also made and recorded in field notes.	Three domains with underlying themes were identified: 1. Challenges of getting treatment and care (service availability, distance/geographic location, transportation, provider collaboration/coordination, physical and emotional safety) 2. Opportunities to bond (proximity, information) 3. Importance of relationships (respect, empathy, familiarity, inclusion, interactions with care providers)
Leiner et al^A12^	“To facilitate greater understanding of perinatal OUD patients' fears, concerns, needs, and priorities and provide lessons for reproductive health and substance use treatment providers”	Sample: 27 perinatal women at a substance use treatment program Setting: United States	Qualitative	Semistructured interviews and focus group were conducted, transcribed, coded, and analyzed for themes.	Three themes were identified: 1. Fears of social services involvement 2. Preparation for delivery 3. Providers addressing fears
Loyal et al^A13^	“To understand the lived experience of nurses on maternity and well-newborn units caring for infants with NAS.”	Sample: 17 postpartum nurses Setting: United States	Qualitative	Focus groups of registered nurses on postpartum units at 2 hospitals were conducted. Themes were identified and the focus groups were stopped when no new themes were identified.	Five major themes were identified: 1. Managing the expectations of parents of newborns with NAS 2. Current NAS protocol (positive aspects of rooming-in and challenges with withdrawal scoring tool) 3. Inconsistencies in care and communication 4. Perceived increase in nursing workload on the postpartum unit 5. Nurses' emotional response to the care of infants with NAS
Maguire et al^A14^	“To learn how caregivers who are expert in feeding infants with NAS successfully feed these infants during withdrawal.”	Sample: 12 total NICU nurses and speech therapists from 3 large regional hospitals Setting: United States	Qualitative	Focus groups were conducted. These groups were recorded and transcribed, and transcriptions were analyzed for content.	Four major themes were identified: 1. Optimal medication management 2. Follow the baby's cues 3. Calm and comfortable 4. Nurture the relationship
McGlothen et al^A15^	“To describe what influences the infant-feeding decisions of women taking medication assisted treatment for an opioid use disorder”	Sample: 8 postpartum women who were receiving medication assisted treatment in the Setting: United States	Qualitative	Individual interviews were conducted, recorded, and transcribed. Transcriptions were then analyzed for content.	Two themes were identified: 1. What I heard about breastfeeding 2. Doing what I feel is best for my baby
McGlothen-Bell et al^A16^	“To explore the skin-to-skin care (SSC) experiences of mothers of infants with NAS, including perceived barriers to SSC in the hospital and following discharge home.”	Sample: 13 mothers with infants with NAS Setting: United States	Qualitative	Semistructured individual interviews were conducted and recorded. Recordings were transcribed and analyzed for content.	Four themes were identified: 1. “A little nerve racking” 2. “She needed me, and I needed her” 3. Dealing with the “hard times” 4. “A piece of my puzzle is missing”
McRae et al^A17^	“To better understand the parent's perspective on the eat, sleep, console (ESC) approach to caring for their opioid-exposed infant during the birth hospital stay.	Sample: 15 mothers and 3 fathers Setting: United States	Qualitative	A content analysis of transcripts from in-depth, semistructured interviews of parents of infants with NAS was conducted. Responses were audiotaped, transcribed, and reviewed by at least 3 members of the research team.	Four major themes were identified: 1. Parents were supportive of fewer interventions and normalizing of newborn care in the ESC approach 2. Parents felt encouraged to lead their infant's NAS care 3. Parents perceived gaps in communication about what to expect in the hospital immediately after delivery and during their infant's hospital stay 4. Parents experienced feelings of guilt, fear, and stress and expressed the need for increased support
Nelson^A18^	“To describe the culture of care and nonpharmacologic nursing interventions performed by NICU nurses for infants with neonatal abstinence syndrome (NAS).”	Sample: 12 nurses Setting: United States	Qualitative, focused ethnography	Individual interviews were conducted, recorded, transcribed, and analyzed for content.	Six themes were identified: 1. Learn the baby (routine care, comfort care, environment, adequate rest and sleep, feeding) 2. Core team relationships (support, interpersonal relationships) 3. Role satisfaction (nurturer/comforter, becoming an expert) 4. Grief 5. Making a difference (wonderful insanity, critical to them) 6. Education and care of the mother
Reese et al^A19^	“To better understand the experiences of nurses and nursing assistants working with women diagnosed with OUD and their newborns “	Sample: 30 nurses and nursing assistants working in a postpartum unit Setting: United States	Qualitative	Four focus groups were conducted. Focus groups were recorded, transcribed, and analyzed for content.	Three themes were identified: 1. Negative feelings and reactions toward patients 2. Preferential concern for the newborn over maternal well-being 3. Identification of organizational and training needs to overcome these challenges
Rockefeller et al^A20^	“To describe mothers' experiences of, supports for, and barriers to bonding with infants with NAS”	Sample: 13 mothers of infants with NAS Setting: United States	Qualitative	Semistructured individual interviews were conducted and audio-recorded. Recordings were then transcribed and analyzed for content.	One overarching theme was identified: 1. Trying to do what is best Four subthemes were identified: 1. Mothers loving their infants and bonding 2. Feeling supported by the infants' fathers 3. Feeling supported in the community 4. Receiving information from hospital staff were associated with mothers' trying to do what is best
Shuman et al^A21^	“To describe perinatal and pediatric nurse perceptions of (1) engaging mothers in the care of opioid-exposed infants and (2) facilitators and barriers to maternal engagement.”	Sample: 21 nurses in either a family birth center, inpatient pediatric unit, or NICU Setting: United States	Qualitative	Individual interviews were conducted and recorded. Recordings were transcribed, and transcriptions were analyzed for content.	Five themes were identified: 1. Vulnerability and bias 2. Mother–infant care: tasks versus model of care 3. Maternal factors affecting engagement and implementation 4. Nurse factors affecting engagement and implementation 5. Recommendations and examples of nursing approaches to barriers
Shuman et al^A22^	“To examine perinatal, neonatal, and pediatric nurse's perceptions regarding the contextual barriers and facilitators related to maternal engagement in and delivery of nonpharmacologic care of neonates with neonatal opioid withdrawal syndrome (NOWS)”	Sample: 21 nurses in a family birth center, inpatient pediatric unit, or NICU Setting: United States	Qualitative	Semistructured individual interviews were conducted and recorded. Recordings were then transcribed and analyzed for content.	Four themes were identified: 1. Lack of education and resources provided to staff and mothers 2. Importance of interdisciplinary and intradisciplinary care coordination 3. Flexibility in nurse staffing models for neonatal opioid withdrawal syndrome 4. Unit architecture and layout affect maternal involvement

ESC, Eat, Sleep, Console; NAS, neonatal abstinence syndrome; NICU, neonatal intensive care unit; OUD, opioid use disorder; SUD, substance use disorders.
